# Complete Genome Sequence of an Emergent Recombinant GII.P16-GII.2 Norovirus Strain Associated with an Epidemic Spread in the Winter of 2016-2017 in Hong Kong, China

**DOI:** 10.1128/genomeA.00343-17

**Published:** 2017-05-18

**Authors:** Martin C. W. Chan, Kirsty Kwok, Tin-Nok Hung, Lok-Yi Chan, Paul K. S. Chan

**Affiliations:** a Department of Microbiology, Faculty of Medicine, Chinese University of Hong Kong, Hong Kong, China; b Stanley Ho Centre for Emerging Infectious Diseases, Faculty of Medicine, Chinese University of Hong Kong, Hong Kong, China

## Abstract

A new recombinant norovirus, GII.P16-GII.2, emerged in the winter of 2016-2017. Here, we report the complete genome of this strain (Hu/GII/HK/2016/GII.P16-GII.2/CUHK-NS-1082), which was collected from a patient hospitalized with gastroenteritis in September 2016 in Hong Kong, China, and sequenced using next-generation sequencing. This genome had a 95.2% nucleotide identity to the closest sequence in GenBank.

## GENOME ANNOUNCEMENT

Human noroviruses comprise a diverse group of enteric RNA viruses that cause a majority of acute gastroenteritis in all age groups worldwide (1). These viruses are genetically very diverse and are currently classified into at least 30 genotypes based on viral capsid protein sequences (2). Genogroup II genotype 4 (GII.4) strains have been predominant since 2002, and new epidemic variants have emerged every 2 to 3 years (3). In the past 3 years, novel non-GII.4 viruses are continuing to emerge, including a new GII.17 Kawasaki variant in the winter of 2014/2015 and a recombinant GII.P16-GII.2 variant in the winter of 2016-2017 (4). Here, we report the complete genome of a GII.P16-GII.2 strain that caused an epidemic spread in the winter of 2016-2017 in Hong Kong, China.

This recombinant norovirus strain (GII/Hu/HK/2016/GII.P16-GII.2/CUHK-NS-1082) was detected as part of our norovirus surveillance (5) in a stool specimen collected from an 8-year-old boy hospitalized for acute gastroenteritis in September 2016 in Hong Kong, China. Preliminary virus capsid genotyping was performed as previously described (6). The complete genome of the virus was determined using a metagenomic next-generation sequencing approach. Briefly, 10% (wt/vol) stool suspension in phosphate-buffered saline was filtered and digested with a cocktail of DNase, RNase, and benzonase to remove cell-free, unprotected host and microbial DNA and RNA. Viral RNA protected in intact virions was extracted and purified using the Viral RNA mini kit (Qiagen). Reverse transcription was performed using tagged random octamers and SuperScript III reverse transcriptase (Thermo Fisher). Second-strand cDNA was synthesized by Klenow fragment (TaKaRa), followed by random cDNA amplification using high-fidelity Phusion Hot Start II DNA polymerase (Thermo Fisher). The library for next-generation sequencing was prepared using the Nextera XT DNA library prep kit (Illumina). Purification between steps was performed using AMPure magnetic beads (Beckman Coulter, Inc.). Paired-end 2 × 75-bp sequencing was performed on the MiSeq system (Illumina). Protocols are available upon request.

A total of 1,025,446 reads were generated. Quality trimming of reads was performed using Trimmomatic version 0.36, and the survival rate was 97.4%. Reference sequence mapping to a recent GII.2 strain (Hu/GII.2/Miyahi1_2012_JP; GenBank accession no. LC145787) was performed on Geneious version 9.1.6, and the percentage of on-target reads was 66.7%. The mean sequencing depth was 6,762× (range 1 to 38,477×). The terminal 5′ and 3′ ends of the virus genome with low coverage (<3×) were validated by Sanger sequencing.

The complete genome of the recombinant GII.P16-GII.2 was 7,536 nucleotides in length, excluding the poly(A) tail. The best BLAST hit in GenBank was a norovirus GII.2 strain collected in 2011 from the United States (Hu/GII.2/HS255/2011/USA; KJ407074) with a 95.2% identity on the nucleotide level. Interestingly, open reading frame 1 (ORF1) of this new GII.2 variant had the highest pairwise identity (98.5% on the nucleotide level and 99.4% on the amino acid level) to a circulating recombinant GII.P16-GII.4 Sydney 2012 strain (Hu/USA/2015/GII.P16_GII.4_Sydney/CA3477; KX907727). It would be interesting to postulate that this emergent GII.2 originated from a recombination event between a GII.4 and a GII.2 virus. Furthermore, this new GII.2 strain contained a one-to-two-codon insertion in the N-terminal protein of ORF1 compared with older GII.2 strains. The functional importance of these insertions merits further investigation.

### Accession number(s).

The complete genome sequence of the recombinant norovirus GII.P16-GII.2 strain (GII/Hu/HK/2016/GII.P16-GII.2/CUHK-NS-1082) has been deposited in GenBank under the accession number KY771081.
